# Antibiotic resistance and preventive strategies in foodborne pathogenic bacteria: a comprehensive review

**DOI:** 10.1007/s10068-024-01767-x

**Published:** 2025-01-29

**Authors:** Masooma Farrukh, Ayesha Munawar, Zeenat Nawaz, Nazim Hussain, Ahmer Bin Hafeez, Piotr Szweda

**Affiliations:** 1 Center for Applied Molecular Biology (CAMB), University of the Punjab, Lahore, Pakistan; 2 Department of Pharmaceutical Technology and Biochemistry, Faculty of Chemistry, Gdańsk University of Technology, Ul. G. Narutowicza 11/12, 80-233 Gdańsk, Poland

**Keywords:** Antibiotic resistance, Foodborne pathogens, Resistance mechanisms, Surveillance strategies, Preventive guidelines, Novel approaches

## Abstract

Antibiotic resistance in foodborne bacteria poses a substantial global health challenge. Reports indicate that antibiotic overuse in middle-class and low-income countries is a significant factor in the ever-increasing resistance. Resistance mechanisms have developed through enzymatic hydrolysis, reduced membrane permeability, efflux pumps, and target site mutations. Preventive measures like proper hygiene and safe food preparation, vaccination, antibiotic stewardship and surveillance, implementing infection prevention and control (IPC) measures, good agricultural practices, and investigating novel approaches like CRISPR, NGS, nanotechnology, and bacteriophages may be employed to address this challenge. Naturally occurring preservatives (e.g., nisin) are alternatives to antibiotics for food preservation. Prebiotics, probiotics, nanobiotics, phage treatment, and antimicrobial peptides are also substitutes for antibiotics. Furthermore, plant-derived compounds, such as essential oils and plant extracts, are promising substitutes for antibiotics in animal production. This review focuses on the mechanisms of underlying antibiotic resistance in foodborne pathogens, necessary preventive measures, and the challenges associated.

**Graphical abstract:**

Created using BioRender https://www.biorender.com/

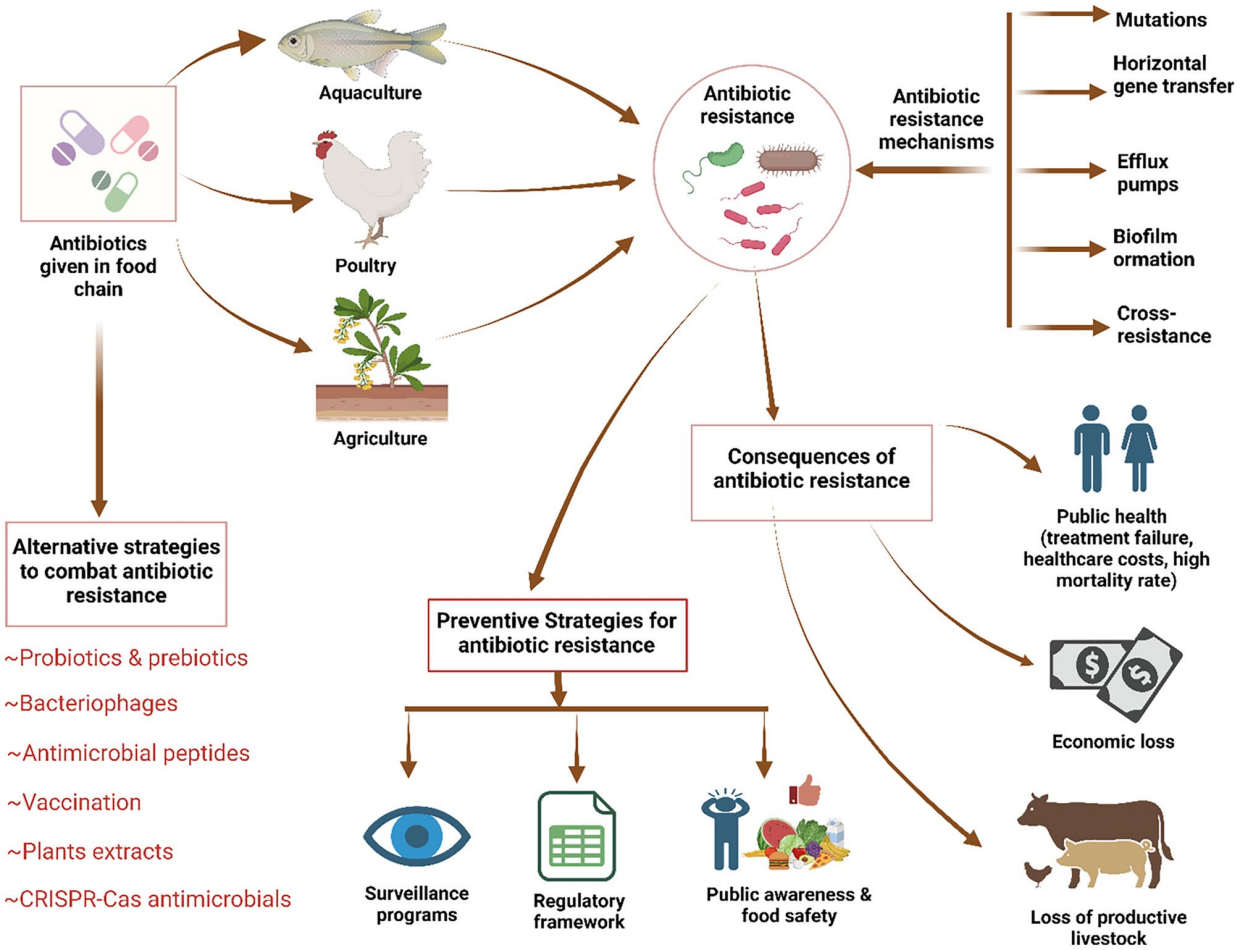

## Introduction

Foodborne illnesses are caused by biological organisms referred to as foodborne pathogens. These include viruses, bacteria, or parasites that are found in food or water and can lead to serious illnesses like food poisoning (Bintsis, 2017; Madilo et al., 2023). Some pathogenic bacteria associated with foodborne illnesses include *Escherichia coli* (found in meat and un-pasteurized milk), *Clostridium botulinum* (found in improperly canned foods), *Campylobacter* (found in raw or undercooked foods), *Salmonella species* (found in meat, poultry, or eggs), *Shigella*, *Clostridium perfringens*, *Vibrio vulnificus*, *Vibrio parahaemolyticus*, *Staphylococcus aureus*, some *Bacillus* species, and *Listeria monocytogenes* (found in undercooked meats, vegetables, unpasteurized milk, and soft cheese) (Havelaar et al., 2015). Unsafe foods that include chemicals and biological toxins are a major cause of sickness and mortality globally (Madilo et al., 2023). All of the major foodborne pathogens, including *Campylobacter* spp., *S. aureus*, *Salmonella* spp.*,* and *L. monocytogenes,* have been shown to exhibit antibiotic resistance and multidrug resistance, which poses a serious threat to public health (Grudlewska-Buda et al., 2023b; Rajaei et al., 2021). Antimicrobial-resistant (AMR) foodborne bacteria can arise and propagate from farm to table by a variety of processes, such as metabolic rearrangement, enzymatic degradation, efflux pump system, target-site mutation, and self-preservation (Kim and Ahn, 2022). Antimicrobial resistant foodborne bacteria are more prevalent in food processing units. There are several times when this contamination might happen, such as while handling, storing, and preparing (Verraes et al., 2013). In order to comprehend the propagation of resistance and provide pertinent risk assessment data, antimicrobial resistance in the food chain must be regularly monitored. A number of studies conducted in numerous countries have shown that bacteria resistant to antibiotics are frequently present in a variety of meats, such as lamb, beef, hog, and chicken. According to a study in Iran, the percentage of uncooked kebab and hamburger samples contaminated with *S. aureus*, *E. coli*, *Salmonella*, and *L. monocytogenes* was 35.5, 8.1, 4.8, and 1.6%, respectively (Rajaei et al., 2021).

In this review, we aimed to provide a comprehensive overview of antibiotic resistance, including its transmission pathways, classifications, and preventive strategies against AMR foodborne pathogenic bacteria. It provides a better understanding of the acquisition mechanisms of antibiotic resistance in foodborne pathogens, factors contributing antibiotic resistance, the effects of antibiotic resistance on public health and the economy, descriptions of antibiotic stewardship programs in food production, alternate approaches to combat antibiotic resistance, and complications in addressing antibiotic resistance in the food chain.

## Trends in antibiotic resistance acquisition over time

Antibiotics are essential to contemporary medicine. Since discovery, they have not only aided in the management of illnesses, but have additionally facilitated the advancement, routine use of invasive surgical techniques and sophisticated medical interventions, such as the care of premature newborns, organ transplants, and cancer treatment (Cecchini and Lee, 2017). In the past, bacteria have shown the ability to become resistant to new antibiotics within a few years of their discovery (OECD, 2018). According to the UK government-commissioned review, AMR might kill 10 million people annually by 2050 (O’neill, 2014, 2016). Yang et al. (2020) analyzed data from eight countries and identified common genes with high frequency of antibiotic resistance. Focusing on the common antimicrobial resistance genes that arise every year for each country and the historical profiles of AMR gene occurrence that are more frequent from 2010 to 2019 they found reported that there is a general increase in the number of cases of antibiotic resistance in foodborne pathogens (Yang et al., 2020).

Similarly, Schar et al. (2021), in their study reported that a major concern is increasing antibiotic resistance in foodborne bacteria. These foodborne pathogens exhibited the highest resistance to tetracyclines (21.5%), sulfonamides (32.9%), macrolides (34.2%), and penicillins (60.4%). The study also revealed high mean resistance rates to the most vitally important and high priority antimicrobials for human medicine, including quinolones, macrolides, and third- and fourth-generation cephalosporins (Schar et al., 2021). As reported in a study by the European Medicines Agency, the anticipated resistance proportions increased by an average of 2% points for 12 combinations of antibiotics and bacteria between 2009 and 2019. *Acinetobacter baumannii* resistance to fluoroquinolone showed the biggest anticipated increases in resistance proportions, while *methicillin-resistant S. aureus* showed the largest projected declines (OECD, 2023). Excessive use of antibiotics promotes the transfer of resistance genes between microorganisms and can spread to pathogens in addition to exerting selective pressure on antibiotic resistance (Juhász et al., 2021; Laxminarayan et al., 2013). According to the World Health Organization (WHO), some foodborne bacteria that cause common infections in the community are becoming more resistant to treatment, and there are high levels of resistance among bacteria that cause potentially fatal bloodstream infections. According to a study, foodborne pathogens' antibiotic resistance shifted toward first declining and then increasing between 2000 and 2020. Overall, the incidence of food isolate pathogen resistance declined from 75% before 2010 to 72% between 2011 and 2015, and then to 80% between 2016 and 2020 (Tao et al., 2022).

## Common foodborne pathogens and their antibiotic resistance profiles

The most often found foodborne pathogens of animal origin that have been detected worldwide include *Staphylococcus*, *Campylobacter*, *Salmonella* and *E. coli* (Rafiq et al., 2022). These bacteria have the potential to cause foodborne illnesses when undercooked food is consumed or when they contaminate food and water supplies (Rafiq et al., 2022). Some foodborne pathogens and their antibiotic resistant rates are given in the Table 1 (Elhadidy et al., 2020; Ford et al., 2023; Grudlewska-Buda et al., 2023a; Kim and Ahn, 2022; Rafiq et al., 2022; Shen et al., 2018).

**Table 1:** Foodborne pathogens and their phenotypic antibiotic resistance profiles in both poultry and livestock food products & by-products

S. nos	Pathogens	Antibiotic	Resistance levels (%)	References
1	*Escherichia coli*	Penicillin	100	Rafiq et al. (2022)
		Tetracycline	72–100	
		Oxytetracycline	78–93	
		Sulfamethoxazole-trimethoprim	51–88	
		Ampicillin	89.5–100	
		Amoxicillin	92–95	
		Streptomycin	19–70	
		Erythromycin	89	
		Ciprofloxacin	50	
		Chloramphenicol	43–50	
		Gentamicin	8–28	
		Enrofloxacin	55	
		Norfloxacin	50	
		Cloxacillin	20	
2	*Staphylococcus aureus*	Penicillin	98.5	Rafiq et al. (2022)
		Amoxicillin	96.5	
		Ampicillin	94.5	
		Cloxacillin	90.5	
		Erythromycin	88.5	
		Gentamicin	29.5	
		Tetracycline	20	
		Ciprofloxacin	12.5	
		Chloramphenicol	10	
		Norfloxacin	10	
		Sulfamethoxazole-trimethoprim	10	
		Enrofloxacin	5.5	
		Kanamycin	5.5	
		Neomycin	5.5	
		Streptomycin	5.5	
		Gentamicin	4.5	
		Tobramycin	4.5	
		Netilmicin	1.5	
3	*Salmonella*	Penicillin	96.15	Rafiq et al. (2022)
		Ampicillin	91.48	
		Oxytetracycline	81.58	
		Sulfamethoxazole-trimethoprim	66.67	
		Gentamicin	3.85	
4	*Campylobacter*	Ciprofloxacin	24	Elhadidy et al. (2020), Ford et al. (2023), Grudlewska-Buda et al., (2023a), Kim and Ahn (2022), Rafiq et al., (2022) and Shen et al. (2018)
		Azithromycin	29	
		Tetracycline	44.5–49.7	
5	*Clostridium botulinum*	Chloramphenicol	10	Swenson et al. (1980)
		Tetracycline	10	
		Fluoroquinolones	15–10	
		Metronidazole	10	
		Trimethoprim- Sulfamethoxazole (SXT)	< 10	
		Nalidixic Acid	< 10	
6	*Aliarobacter* spp.	Tetracycline	78	Grudlewska-Buda et al. (2023b)
		Ciprofloxacin	24–59	
		Beta-lactamases	< 1	
		Ampicillin	56	
		Macrolides	71	
		Chloramphenicol	19	
7	*Vibrio* spp.	β-lactams	High resistance	Grudlewska-Buda et al. (2023b)
		Tetracycline	29	
		Sulfonamide	High resistance (% not mentioned)	
		Aminoglycosides	Low resistance	
		Macrolides	Moderate resistance	
		Chloramphenicol	Low resistance	
8	*Listeria monocytogenes*	Ciprofloxacin	1.8	Grudlewska-Buda et al. (2023b)
		Tetracycline	8.4	
		Vancomycin	Low to moderate resistance	
		Trimethoprim		
		Lincomycin		
		Macrolides		
		Sulfonamides	73	
9	*Shigella* spp.	Tetracycline	Highest (up to 62%)	Tao et al. (2022)
		Fluoroqunilones	High	
		Aminoglycosides	High	
		Beta-lactamases	High (62%)	
		Chloramphenicol	Low	
		Sulfonamide	High	
10	*Streptococcus suis*	Clindamycin	6.5	Grudlewska-Buda et al. (2023b) and Kim and Ahn (2022)
		Tetracycline	44.35–66	
		Doxycycline	9.2	
		Penicillin	Moderate resistance	
		Macrolides	Moderate resistance	
		Aminoglycosides	Low resistance	

## Factors contributing to acquisition of antibiotic resistance in foodborne pathogens

The demand for meat has grown over the past few decades, leading to an increase in worldwide meat production, which may be related to the rise in antibiotic usage. By 2030, the amount of antibiotics consumed worldwide might rise to 110,000 tons (Aarestrup, 2015). The improper and excessive use of antibiotics in human medicine and food production, together with bacteria's innate ability to adapt and become resistant over time, are some of the causes of antibiotic resistance in foodborne pathogens (Bilal et al., 2017). According to the study, foodborne bacteria isolated from poultry have a higher phenotypic resistance to antibiotics than those from other sources like sewage water, seafood or dairy products (Rafiq et al., 2022). This is in line with other research that foodborne bacteria (*E. coli*, *Salmonella* spp*.*, *L. monocytogenes*, *S. aureus*, *C. botulinum*) isolated from poultry products have significant levels of antibiotic resistance. Multidrug resistance was present in a significant amount of poultry *E. coli* strains (62.5%) of the bacteria in chicken samples (Mensah et al., 2022). Another factor causing antibiotic resistance in foodborne bacteria is the wide spread of resistance conferring genes up the food chain (Rafiq et al., 2022). A study suggests that enterococci and streptococci are the common sources of resistance genes for foodborne pathogens like *L. monocytogenes* (Rafiq et al., 2022). Following are the major factors that are contributing to antibiotic resistance in foodborne pathogens.

## Use of antibiotics in agriculture and aquaculture

Antibiotic-resistant bacteria have been found to arise as a result of antibiotic use in agriculture and aquaculture can then spread to humans through food chains and the environment Bilal et al. (2017, 2019, 2020a; Economou and Gousia, 2015; Manyi-Loh et al., 2018). Significant differences exist in the patterns of antibiotic consumption in agriculture between developing world regions and nations, and the majority of developing nations continue to use antibiotics that are prohibited in other nations, including industrialized nations (Adebowale et al., 2016; Moyane et al., 2013). It was anticipated that the antibiotic usage will roughly treble among the BRICS nations, which include Brazil, Russia, India, China, and South Africa (Van Boeckel et al., 2015). The estimate stems from the move to large-scale farming that requires frequent use of antibiotics in order to keep livestock healthy and productive. Concerningly, the classes of antibiotics used in veterinary and agricultural practices usually have same modes of action and are closely connected or similar to those given to humans (Islam et al., 2016).

The persistence of these superfluous antibiotics in animal husbandry could be attributed to several factors, including the expansion and concentration of farmlands, insufficient government regulations and supervision regarding the use and distribution of antibiotics, restricted application of infection control techniques, and farmers' reluctance to execute assigned modifications in farming practices (Kaplan et al., 2014). Since most antibiotics are not completely metabolized and are released into the environment as waste products, which have the potential to change the population of bacteria and increase antibiotic resistance, it has an ecological impact. Antibiotics that end up in aquatic habitats due to human pressure may have an impact on the microbial communities. By altering the makeup of bacterial communities, preventing or enhancing their ecological roles, and enhancing and sustaining drug resistance, antibiotics are ecological variables that propel microbial evolution (Kulik et al., 2023). Several studies reported the prevalence of antibiotic resistance in *Acinetobacter baumannii* found in diverse environmental specimens (Zhu et al., 2013) and found that manure and soil samples from three large commercial swine farms in three different regions contained notable concentrations of tetracycline. A broad spectrum of antibiotic resistance genes (149 unique ARGs) were also found and it was noted that 43% of the aminoglycoside phosphorylation gene *aphA3* was present in all manure samples (Xiao et al., 2016). Numerous genes related to antibiotic resistance were found in the paddy soil genome sequencing results. Multidrug resistance was the most prevalent, present in 38–47.5% of the soil samples under evaluation.

Antibiotics are used in aquaculture for preventative, therapeutic, metaphylactic, and growth-promoting reasons (Okeke et al., 2022; Pepi and Focardi, 2021). Antibiotic-resistant microorganisms reach aquatic environments via human and animal sources. These antibiotic-resistant microbes have the ability to transfer their genes to aquatic microorganisms and induced the antibiotic resistance in them (Kraemer et al., 2019). The most commonly used antibiotics in aquaculture are oxytetracycline, florfenicol, and sulfadiazine (Okeke et al., 2022; Pepi and Focardi, 2021) which gives rise to antibiotic-resistance, subsequently transmitting it to clinically relevant strains, impacting the entire ecosystem (Preena et al., 2020).

To prevent this spread in aquaculture, continuous monitoring, early identification of resistant strains, and adequate restrictions are essential (Preena et al., 2020). Moreover, a better understanding of gene transfer mechanisms, such as plasmids, transposons, integrons, and gene cassettes, is required to unravel the complicate process of antibiotic resistance in aquaculture (Preena et al., 2020). For the aquaculture sector, antibiotics are frequently provided as feed or by absorption in enclosed vessels (Fang et al., 2019). A fraction of food treated with antibiotics that the fish do not consume is accumulated in the sand dunes around and beneath aquatic farming sites (Aarestrup, 2006; Cabello et al., 2013; Sapkota et al., 2008; Sarmah et al., 2006). Examinations of the antibiotics oxytetracycline, florfenicol, and flumequine in the seabed underneath Greek fish farms in the Eastern Mediterranean Sea revealed that flumequine was the only antibiotic that could be identified, though in relatively small concentrations. It indicates that antibiotic resistance exists there, even in minor doses (Kalantzi et al., 2021). 90% of extended-spectrum β-lactamase-producing Enterobacteria (ESBL-E) in aquatic environments are resistant to one antibiotic or more, and 20% are resistant to multiple antibiotics (Woerther et al., 2013). Multi-resistant bacteria can arise when several antibiotics are used simultaneously in aquaculture. The consumption of raw or undercooked seafood contaminated with antibiotic-resistant bacteria, improper handling, processing, and preparation techniques for aquatic products, and other factors can all contribute to the transmission of antibiotic-resistant bacteria from aquaculture into the food chain. Moreover, the improper disposal of trash can lead to the contamination of water bodies by antibiotic-resistant bacteria and resistance genes originating from aquaculture (Perreten, 2014).

## Cross-contamination in food processing and handling

Foodborne bacteria may develop antibiotic resistance due to cross-contamination during manufacturing and handling. Feces from the slaughter process can contaminate animal products with microorganisms resistant to antibiotics, while the use of fertilizers or irrigation water during the production process can contaminate plant products (Verraes et al., 2013). During handling by the consumer, food might become contaminated with antibiotic resistant bacteria or genes from another food item in food preparation area. This is known as cross-contamination (Samtiya et al., 2022; Verraes et al., 2013). The transmission of bacteria from raw to ready-to-eat foods, such as *E. coli* and *Campylobacter*, can result in cross-contamination and food poisoning. Cross-contamination can also result from inappropriate food storage practices and the usage of chopping boards and utensils for raw meat. Microorganisms that may possess antimicrobial resistance genes are purposely introduced to certain food items during the manufacturing process for technical purposes (Verraes et al., 2013). Cross-contamination can occur when microorganisms harboring antibiotic resistance genes, especially those carried by phages, are purposefully added to food during processing. This may contribute to spread antibiotic resistance. These microorganisms are categorized into four types based on their intended effect: starter cultures, probiotics, bacteriophages and bio-preserving microorganisms (BRI, March 2016).

Tetracycline resistance is more common than transferable resistance genes when it comes to antimicrobial resistance in beginning cultures, according to a typical result (Authority, 2008). Antibiotic resistance has occasionally been connected to fermented foods and probiotic bacteria. It is possible for probiotic bacteria to transmit genes that confer resistance to antibiotics via horizontal gene transfer processes such conjugation, transformation, and transduction. The genes could be linked to transposons, integrons, and other mobile genetic elements that promote inter-bacterial transfer. Interactions within the gut microbiome, stress-induced circumstances, and antibiotic-induced selective pressure can all have an impact on the transfer. (Masco et al., 2006; Teuber et al., 1999). Probiotic *Bifidobacteria,* such as *Bifidobacterium animalis* subsp. *lactis* and *Bifidobacterium bifidum,* have also been found to be resistant to tetracycline (Masco et al., 2006). In Enterococcus isolated from naturally fermented foods, tetracycline resistance is the most prevalent kind of antibiotic resistance According to a study, 89.5% and 53% of Enterococcus isolates from conventional chicken and beef samples, respectively, were resistant to tetracycline (Yılmaz et al., 2016).

## Globalization and international trade

Antibiotic resistance in foodborne pathogens is significantly impacted by globalization and international trade. Antibiotic resistance is mostly disseminated via the international commerce in cattle, food animals, and their feed, especially in animals that have resistant bacteria (Hanefeld et al., 2017). For instance, drug-resistant *E. coli* may survive in minced beef that has been kept for many days as well as on beef carcasses in a chiller for up to 24 h (Alexander et al., 2010). According to a study submitted by retail dealers in the Netherlands many of the uncooked meat samples had methicillin-resistant *S. aureus* (MRSA), ranging from 2.2% of animal samples to 35.3% of turkey samples (De Boer et al., 2009). Antibiotic resistance is also facilitated by trade agreements and legislation. In 2006, the European Union (EU) prohibited the use of antimicrobials for the purpose of promoting growth in livestock production systems (Wallinga et al., 2022). However, this policy change led to a 'repackaging' of the products' labeling and marketing, positioning them as 'prophylactic therapeutics' rather than 'growth promoters'. Using antibiotics as growth promoters increases animal growth and feed conversion while lowering morbidity and death from both clinical and subclinical illnesses (Butaye et al., 2003).

## Acquisition mechanisms of antibiotic resistance

Given the widespread outbreak of antibiotic resistance, there are two different proposed mechanisms of antibiotic resistance, (i) genetic mechanisms, and (ii) phenotypic mechanisms (Hashempour-Baltork et al., 2019; Yi and Ahn, 2023).

## Genetic acquisition mechanisms

Within bacterial species, there is a complex interaction between innate and acquired variables (horizontal gene transfer, efflux pumps and mutations) that contribute to the genetic processes of antibiotic resistance. Through a variety of genetic components and processes, these pathways may contribute to the development of resistance.

**Fig. 1:**
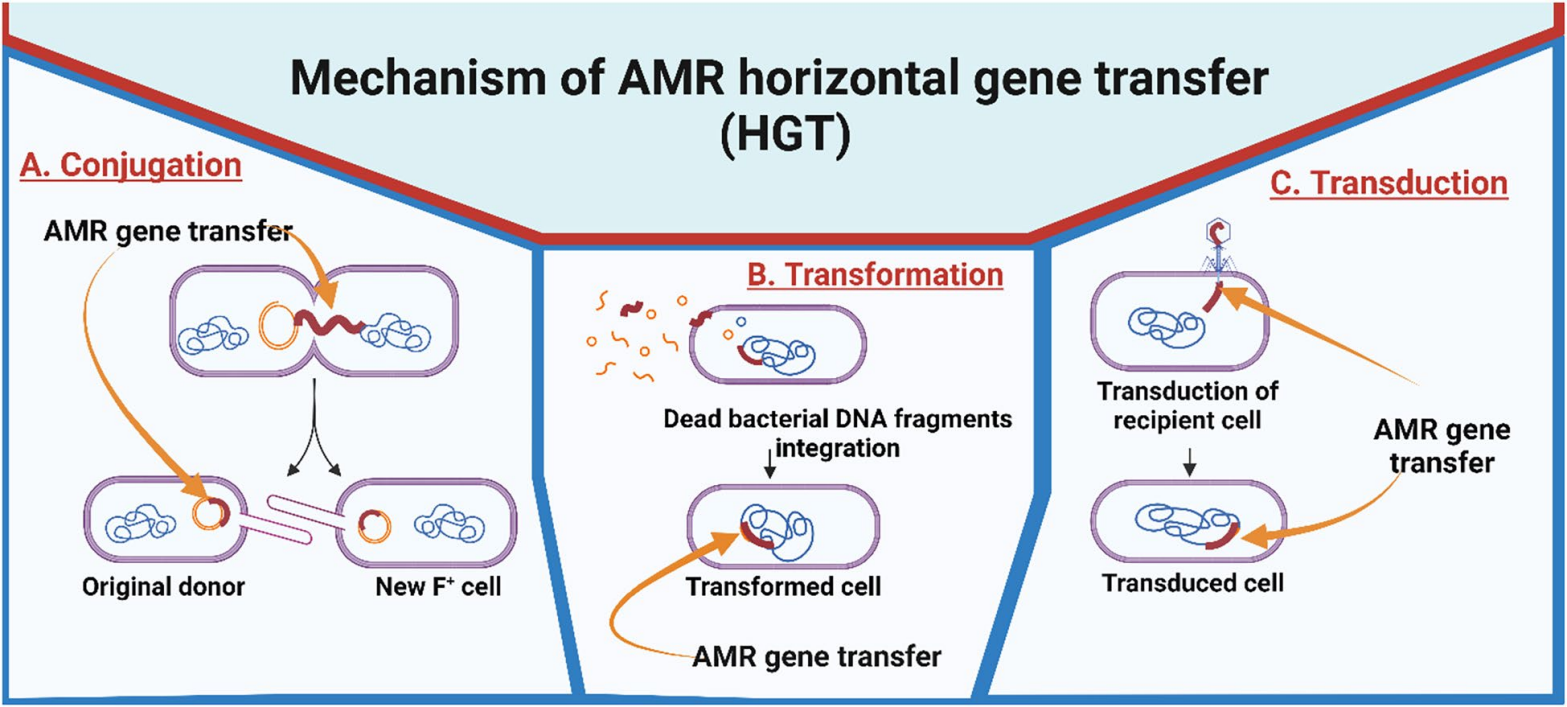
Horizontal gene transfer. Conjugation, transformation and transduction are the basic processes involved in antimicrobial resistant horizontal gene transfer (HGT) which is a type of genotypic mechanism of antibiotic resistance. Created using BioRender https://www.biorender.com/

The three basic processes of horizontal gene transfer (HGT) among bacteria are conjugation, transformation, and transduction (Fig. 1) (Verraes et al., 2013). This method makes it possible for resistance characteristics to spread quickly throughout various bacterial populations, which causes multidrug-resistant "superbugs" to form and pose serious risks to the health of people and animals (Sun et al., 2019). Antimicrobial resistance genes can also be linked to genomic islands, insertion elements, and transposons.

Mutations that result from exposure to antibiotics or from spontaneous occurrences can lead to antibiotic resistance in bacteria (Hasan et al., 2022). It was shown in the 1980s that some antibiotics cause higher than normal rates of bacterial mutation. This led to the proposal of the "increased evolvability" theory, which states that bacterial populations' genetic diversity is increased by antibiotic-induced mutagenesis, speeding up the growth of antibiotics resistance bacterial (Vasse et al., 2022). Mutation is often main cause of resistance to fluoroquinolones (Ruiz, 2003), rifampicin (Gillespie, 2002), oxazolidinones (Long and Vester, 2012), fusidic acid (Besier et al., 2005), and streptomycin (Gillespie, 2002). In the most basic case, mutations occurred during DNA synthesis resulted in spontaneous mutations which leads to antibiotic resistance in bacteria (Martinez and Baquero, 2000). Antibiotic exposure might increase the rate of bacterial mutation. Antibiotic exposure can increase the rates of mutation directly by either temporarily downregulating mismatch repair or by activating error-prone DNA repair pathways of bacteria. (Shee et al., 2011).

## Phenotypic acquisition mechanisms

Antibiotic resistance phenotypic mechanisms are linked to certain situations such as growth arrest, modifications in bacterial metabolism, and differences in antibiotic sensitivity depending on the metabolic status of bacterial populations (Kok et al., 2022). Comprehending these pathways of phenotypic resistance is essential for creating innovative therapeutic strategies that take into account the true susceptibility of bacteria, which may vary from conventional laboratory assessments (Corona and Martinez, 2013).

Even though planktonic bacteria (*E. coli*, *L. monocytogenes*, *Vibrio* spp., *S. aureus*, *Streptococcus* spp., *Campylobacter* spp., *Bacillus cereus*) are used in the majority of investigations on antibiotic sensitivity in bacteria, microbes may proliferate and form biofilms when they adhere to a surface (Costerton et al., 1999; Hall-Stoodley et al., 2004; Hansen et al., 2007; Kolter and Greenberg, 2006). Biofilms are intricate formations that make it more difficult for antibiotics to diffuse freely. During biofilm growth, collective activities are coordinated by a system of cell-to-cell communication called quorum sensing. Genes that encode extracellular matrix components, polysaccharides, and surface-associated proteins are among those essential for the production of biofilms (Peng et al., 2023). SNPs and other genetic sequence variations can affect how biofilms form. Research has demonstrated a correlation between biofilm forming capacity and SNPs linked to genes such as alpA, alpB, cagE, and csd4. These genetic differences may impact bacterial strains' ability to adhere to surfaces and form biofilms (Fauzia et al., 2023). The antibiotic's structure will determine how well it diffuses into the biofilm (Corbin et al., 2011; Singh et al., 2010). Biofilms exposed to cefotaxime produced less biomass than controls, suggesting that resistance mechanisms have a detrimental effect on biofilm formation. It has been proposed that the bacteria that create biofilms can exhibit a variety of metabolic states, such as rapidly developing cells that ought to be killed by antibiotics and resting cells that might only momentarily withstand them (Wan et al., 2018). Depending on the antibiotic family, various metabolic conditions have varying effects. *Pseudomonas aeruginosa* biofilms have been shown to have aerobic parts that are resistant to cationic peptides and vulnerable to quinolones, whereas the hypoxic sections exhibit the reverse properties like reverse diauxie (in which it utilizes lactic acid in hypoxic situations rather of glucose) and altered nutrients utilization (Rani et al., 2007).

Efflux pumps play a significant role in the development of antibiotic resistance in bacteria. Bacterial membrane proteins known as efflux pumps actively move materials including antibiotics out of the cell, which greatly contributes to the emergence of antibiotic resistance. The extrusion of hazardous substrates from bacterial cells, including almost all kinds of therapeutically relevant antibiotics, is facilitated by these transport proteins (Webber and Piddock, 2003). There are five main families of efflux transporters in the bacterial kingdom: MATE (multidrug and toxic efflux), RND (resistance-nodulation-division), SMR (small multidrug resistance), and ABC (ATP binding cassette). The RND (resistance-nodulation-division) is known as major facilitator (Lomovskaya et al., 2001). Mutations in local repressor genes can cause efflux pumps to be overexpressed (Adewoye et al., 2002). The wide range of substrates that efflux systems may handle is concerning since over-expression of a pump can lead to resistance to several classes of antibiotics, as well as to certain dyes, detergents, and disinfectants (including several widely used biocides) (Commission).

**Fig. 2:**
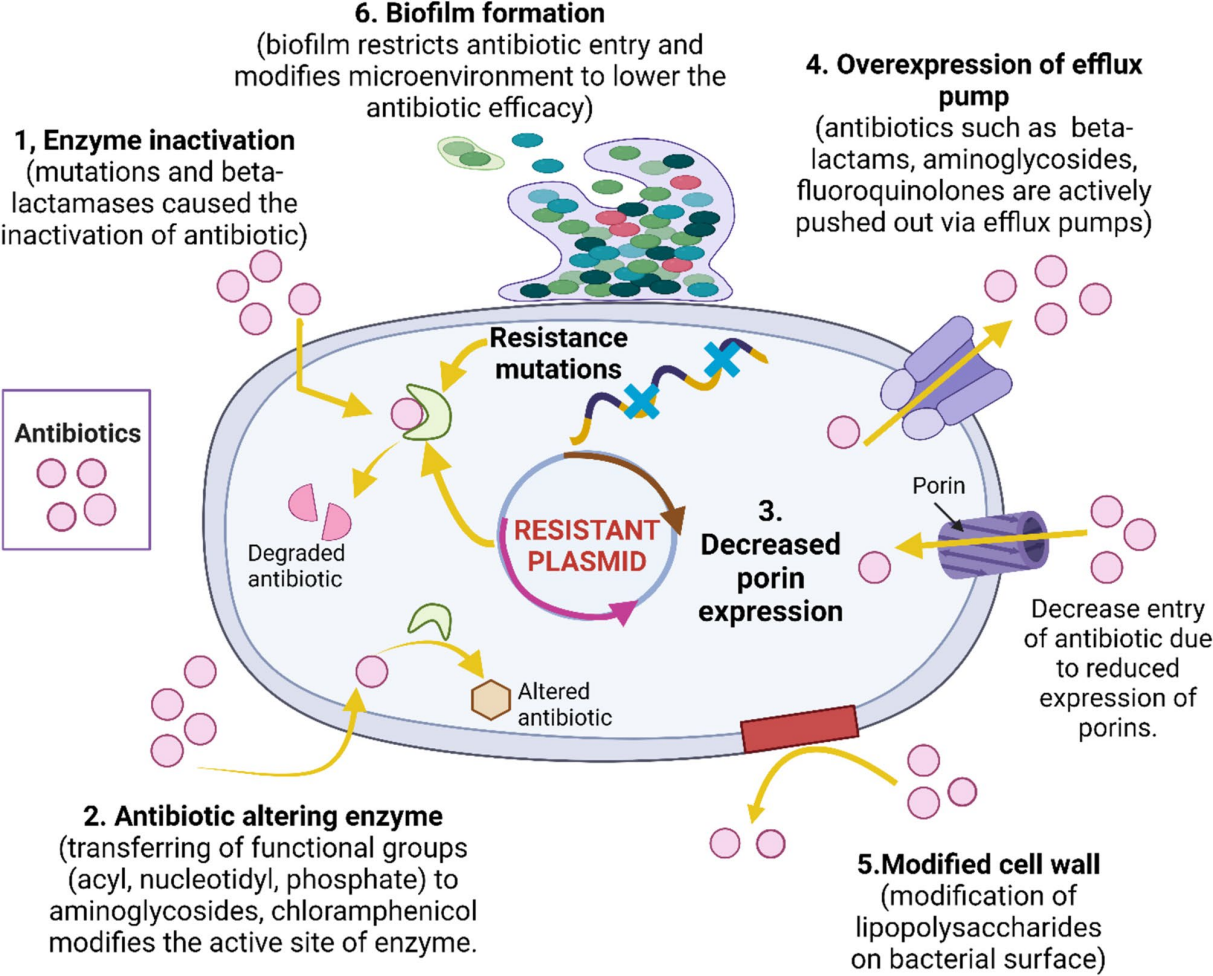
Phenotypic mechanism of antibiotic resistance. Phenotypic mechanisms such as biofilm formation, efflux pumps and transient variation in permeability of bacteria to antibiotics are among the major players responsible for resistance in bacteria. Figure was Created using BioRender https://www.biorender.com/

Six distinct strategies can be used to control an organism's permeability to antibiotics (Fig. 2). (1) Under the strain of antibiotic selection, the enzyme inactivation functions as a self-resistance mechanism. The penicillin-binding proteins (PBPs), which are crucial for the formation of peptidoglycan, share structural similarities with β-lactam antibiotics. Penicillin and cephalosporin, two structural analogs, permanently acylate a PBP's active site, which degrades the antibiotic (Kim and Ahn, 2022). Penicillins, monobactams, and cephems are resistant to mutations that result in hyper-inducible AmpC synthesis or that cause enhanced expression of AmpC enzymes. Furthermore, resistance might range widely due to horizontal acquisition of several β-lactamases from classes A, B, and D. A number of these β-lactamases are connected to specific "high risk" P. aeruginosa clones that are widely dispersed around the world (Eichenberger and Thaden, 2019). (2) Antibiotic resistance in bacteria can be caused by the hydrolase-type enzymes breaking down substrates such aminoglycosides, phenicols, macrolides, and β-lactams. Antibiotic resistance may result from the steric obstruction of antibiotic target sites. By transferring functional groups (acyl, phosphate, nucleotidyl, and ribitoyl) to aminoglycosides, chloramphenicol, streptogramins, and fluoroquinolones, antibiotic-modifying enzymes such transferases can confer antibiotic resistance (Kim and Ahn, 2022). (3) The susceptibility to antibiotics may vary depending on changes in the quantity or type of porins or antibiotic transporters. (4) The antibiotic can actively be pushed outside the cell via efflux pumps after it enters the cytoplasm (Corona and Martinez, 2013). (5) Bacteria can change the lipopolysaccharide on their surface, which disrupts the antibiotic's molecular interactions and, as a result, its penetration. The process of creating membrane vesicles increases the amount of membrane surface that is available, which may also lower the effective concentration of antibiotic that can enter a cell. (6) By establishing a physical barrier that restricts medication penetration and encouraging microenvironments that lower antibiotic efficacy, biofilm development increases resistance to antibiotics. Furthermore, persisted cells reside in biofilms, which promote genetic exchange and hasten the dissemination of resistance characteristics among bacterial populations (Sadık et al., 2020).

When bacteria simultaneously develop resistance to many chemicals, such antibiotics and heavy metals, through comparable processes, this is referred to as co-resistance (Wales and Davies, 2015). In bacterial isolates, co-resistance to heavy metals and antibiotics seems to work in concert through comparable pathways. Antibiotic resistance genes in the environment may be amplified by this synergy and then transmitted to clinical settings (Edet et al., 2023). Genetic connections between metal resistance and antibiotic resistance genes can lead to co-resistance, when the two are inherited together (Murray et al., 2024). The co-location of these genes on mobile elements, including transposons and plasmids, increases the likelihood that bacteria may pass co-resistance (Edet et al., 2023).

The phenomenon known as cross-resistance occurs when bacteria use comparable pathways to gain resistance to a variety of chemicals, including antibiotics. This can happen when bacteria acquire genes that make them resistant to drugs or when they have gene mutations that change how sensitive they are to antibiotics (Cherny et al., 2020). Cross-resistance is an issue when an efflux pump is exposed to a single agent that is part of its substrate profile, it tends to over-express that efflux pump and becomes resistant to all other substrates (Webber and Piddock, 2003).

## Consequences of antibiotic resistance

When compared to identical infections caused by susceptible strains of bacteria, infections produced by antibiotic-resistant bacteria can result in up to two time greater risks of unfavorable consequences (Friedman et al., 2016). Antibiotic resistance poses a risk to treatable diseases like pneumonia, TB, or mild infections, making them incurable and burdening families and the healthcare system more financially and emotionally (Chenoweth et al., 2000).

## Public health consequences of antibiotic resistance in foodborne bacteria

Global public health concerns about antibiotic resistance can have serious repercussions, such as a higher chance of developing a serious, protracted disease or dying from it, experiencing severe drug side effects, needing more frequent checkups and hospital stays, and spending more financial resources on medical treatment. Consuming food or water contaminated with antibiotic-resistant bacteria can result in foodborne sickness in the public. There are around nineteen pathogenic bacteria known to be responsible for outbreaks of foodborne diseases. Following is the table of major foodborne pathogenic bacteria (Table 2) (Nazir et al., 2023).

**Table 2:** Major Foodborne pathogenic bacteria, diseases and food items associated with these bacteria

S. nos	Pathogen	Disease	Commonly associated foods	References
1	*Campylobacter* spp.	Campylobacteriosis	Undercooked poultry, unpasteurized milk, water	Giangaspero et al. (2023)
2	*Shiga toxin–*producing *E. coli* non-O157	Hemolytic uremic syndrome (HUS), diarrhea, vomiting	Undercooked beef, pork, chicken, contaminated fruits and vegetables	Marder et al. (2023) and Rounds et al. (2012)
3	*Brucella* spp.	Brucellosis	Raw unpasteurized dairy, undercooked meat	Qureshi et al. (2023)
4	*Streptococcus* spp. group A (GAS)	Cellulitis, Necrotizing fasciitis, pharyngitis, streptococcal toxic shock syndrome etc.	Unhygienically handled food, e.g. pasta, sandwiches	Brouwer et al. (2023), Falkenhorst et al. (2008) and Okamoto et al. (2014)
5	Enterotoxigenic* E. coli*	Traveler’s diarrhea	Raw vegetables, untreated water, street food	Buuck et al. (2020) and MMWR (1993)
6	*Bacillus cereus*	Food poisoning (diarrheal and emetic syndrome)	Rice, dairy, infant foods	Chen et al. (2022)
7	*Clostridium botulinum*	Botulism	Canned foods, improperly,preserved/stored vegetables (spinach, mushrooms), meat, seafood, water	WHO (2023b)
8	*Staphylococcus aureus*	Bacteremia, infective endocarditis etc.	Raw meat, raw milk, cheese, bakery products etc.	Kadariya et al., (2014) and Tong et al. (2015)
9	*Shiga toxin–*producing *E. coli* O157	Hemorrhagic colitis (HC), hemolytic uremic syndrome (HUS)	Undercooked beef, raw milk, contaminated produce	Etcheverría and Padola (2013)
10	*Vibrio vulnificus*	Vibriosis	Raw/undercooked oysters, seafood	Dutta et al. (2021)
11	*Clostridium perfringens*	Food poisoning, gastroenteritis	Meat, poultry, gravies	Larry (2023)
12	*Vibrio parahaemolyticus*	Vibriosis	Raw/undercooked seafood (oysters, crabs, fish, sushi)	Dutta et al. (2021)
13	*Mycobacterium bovis*	Tuberculosis (TB)	Unpasteurized milk, dairy products	Krajewska-Wędzina et al. (2022)
14	*Shigella* spp.	Bacillary dysentery (Shigellosis)	Raw vegetables, contaminated water, potato salad, tuna salad etc.	CDC (2024) and Lampel et al. (2018)
15	*Listeria monocytogenes*	Listeriosis	Deli meats, soft cheeses, raw milk	Rogalla and Bomar (2024)
16	*Salmonella* spp.	Salmonellosis	Chicken, pork, eggs, fruits and vegetables	Popa and Papa (2021)
17	*Vibrio cholerae*	Cholera	Fish and shellfish	Dutta et al. (2021)
18	*S. enterica serotype Typhi*	Typhoid fever	Dried milk products, milk chocolate, peanut butter, pork, eggs, water etc.	Bhandari et al. (2024) and Ehuwa et al. (2021)
19	*Yersinia enterocolitica*	Yersiniosis	Pork, unpasteurized milk, undercooked meat, contaminated water	Aziz and Yelamanchili (2024) and Bari et al. (2011)

## Increased treatment failure and severity of infection

New diseases and outbreaks have emerged as a result of shifting trends in food production and distribution, such as the advent of contemporary intensive farming and the formation of biofilms (Nazir et al., 2023). Antibiotic-resistant bacteria reduce the effectiveness of drugs intended to eliminate them or inhibit their growth, posing challenges in treating foodborne illnesses caused by them. Sepsis is more likely to occur in cases of antibiotic-resistant bacterial infection (Kumar et al., 2024). Due to the prolong sickness, higher frequency of septicemia, and severe symptoms can lead to expensive treatments, hospital stays, and increased risks of side effects (CDC).

## Higher mortality rates and healthcare costs

The center for disease control (CDC) estimates that foodborne infections cause 3,000 fatalities in the US each year (CDC). Immuno-compromised individuals, the elderly, and young children are frequently more susceptible and have a greater chance of infections (Smith, 1998). Additionally, people having pre-medical complications, foodborne infections can make their life-threatening illnesses worse (Barton Behravesh et al., 2011). In 2003, the yearly cost of disease caused by *E. coli* O157 (O157 STEC), which produces shiga toxin, was $405 million (US dollars). This amount included $370 million for preventable deaths, $30 million for medical expenses, and $5 million for lost productivity (Frenzen et al., 2005).

## Challenges in diagnosis and societal consequences

A foodborne illness's diagnosis and treatment are determined by the patient's medical history and physical examination. Foodborne infections frequently cause vomiting, diarrhea (bloody or not), fever, headaches, abdominal cramps, dehydration, myalgia, and arthralgias (Switaj et al., 2015). Stool cultures, the gold standard for identifying bacterial foodborne illnesses, only provide positive results in fewer than 40% of cases, which presents difficulties in the diagnosis of antibiotic-resistant foodborne illnesses (Switaj et al., 2015). This implies that more testing may be required to establish the presence of antibiotic-resistant bacteria, and that a negative culture result does not always rule out a bacterial foodborne disease. Since there are no particular symptoms associated with foodborne illness, the doctor must make an appropriate diagnosis based on the patient's history, epidemiologic factors, and objective results (Switaj et al., 2015). Foodborne illnesses have a substantial financial cost, reduce productivity, and have an adverse effect on public health. The financial toll that foodborne infections take includes lost productivity from people who are unable to work and medical costs.

## Economic implications for the food industry and society as a whole

The USDA's Economic Research Service (ERS) has been estimating the financial impact of fifteen major foodborne diseases since the mid-1990s. These includes illnesses caused by *Campylobacter*, *Clostridium*, *Cryptosporidium*, *E. coli* O157, *Cyclospora*, *E. coli* non-O157, *Listeria* spp., *Shigella*, *Salmonella*, *Vibrio* spp., *Vibrio parahaemolyticus*, *Vibrio vulnificus and Yersinia* (Hoffmann and Ahn, 2021). A meta-analysis of earlier research led ERS to estimate the worth of each life saved in the US at $8.7 million dollars in 2013, rising to $9.7 million dollars in 2018 after accounting for income growth and inflation. The $2 billion rise in the overall cost of these foodborne diseases between 2013 and 2021 was 76 percent of this $1.6 billion increase (Hoffmann and Ahn, 2021). Foodborne diseases have considerable costs, including medical expenses, loss of productivity, and financial losses for food companies. When foodborne disease outbreaks occur, firms in the food sector may experience lower sales, bad press (negative publicity), reputational harm, increased legal expenses, or perhaps have to close permanently. A decline in customer trust in the food supply's safety can have long-term consequences, including modifications to consumer preferences and behavior (Macready et al., 2020).

## Loss of productive livestock and crops

Antibiotics resistant foodborne infections cause productive livestock and crops to perish because afflicted animals may need long-term care and harvests may need to be thrown out. Treating resistant cattle diseases can also be expensive since it may be necessary to use expensive medications or alternative therapies. Through the food chain, antibiotic-resistant bacteria in cattle can infect people, resulting in complex, incurable, and protracted diseases that can occasionally be fatal (Manyi-Loh et al., 2018). Antibiotic-resistant genes (ARGs) are released into the environments (such as soil and water) through cattle feces with antibiotic resistant strains. The possibility of human exposure would rise with more ARG replication and proliferation, especially for agricultural workers and those residing nearby (Li et al., 2015).

## Antibiotic stewardship in agriculture and food production

A safe food supply and the prevention of antibiotic resistance in animals and humans depend significantly on antibiotic stewardship in agriculture and food production. Without lowering agricultural productivity, antibiotic stewardship in food production has been attributed to lower rates of resistance in humans and animals (Patel et al., 2020). Antibiotic stewardship programmes (ASPs) aim to uphold the efficacy of antibiotics for the benefit of both animal and human health while also promoting their appropriate usage. The American Veterinary Medical Association (AVMA) outlines four pivotal tenets of antibiotic stewardship:

i. A commitment to stewardship.
ii. An emphasis on disease prevention.
iii. The cautious use of antibiotics.
iv. The monitoring and reporting of antibiotic usage and resistance (AVMA).

Through the incentive of certification, animal management certification programmes can encourage compliance with antibiotic stewardship on farms. Programmes like U.S. Poultry and Egg Association, National Dairy FARM Programme, and Beef Quality Assurance have shown an excellent deal of variability in meeting the metrics of the Antibiotic Stewardship Assessment Tool (ASAT) in the US. This is partly because the programmes have different audiences and objectives (Umber and Moore, 2021).

A number of nations, notably those in Europe, have worked diligently to minimize the usage of antibacterial drugs in animals reared for food through implementing an assortment of stewardship measures (More, 2020). Antibiotic usage in the Netherlands is assessed across farms, identifying veterinary prescribers as well as medium, high, and extremely high consumers, with the possibility of regulatory penalties (Speksnijder et al., 2015). The use of antibiotic growth promoters was declared unlawful by the European Union in 2006 (Union, 2022). New regulations, which are scheduled to take effect in 2022, prohibit the use of antibiotics in groups of animals or medicated feeds. These regulations put constraints on the use of metaphylactic antibiotics, bolster the ban on growth-promoting agents, call for member states to gather information on the supply and consumption of antibiotics. These new regulations also stipulate the possibility of reserving certain antibiotics for consumption by humans only, and forbid the importation of meat raised using growth promoting agents (Union, 2022). Decrease in resistance to antibiotics in animals reared for food has been linked to antibiotic stewardship practices on farms. Within the span of two years of Denmark outlawing the use of avoparcin, an antibiotic that is analogous to vancomycin, the prevalence of vancomycin-resistant enterococci in humans and cattle dropped (Levy, 2014).

## Regulation and control of antibiotic usage in animal feed and growth promotion

The excessive consumption of antibiotics has led to a considerable increase in the emergence of microorganisms in the twenty-first century, and these organisms have been shown to regulate their genetic makeup in a faster and more efficient manner, which helps them acquire resistance to multiple antibiotic groups, particularly antibiotics with a broader spectrum. It has been noted that even the latest generation of antibiotics is inadequate against such a variety of microorganisms. This creates an important obstacle for scientists, prompting the development of novel strategies to tackle this recurring issue. Consequently, it is vital that novel strategies be developed in order to reduce or offer a substitute option to this burgeoning issue of multiple drugs resistance (Mann et al., 2021).

**Fig. 3:**
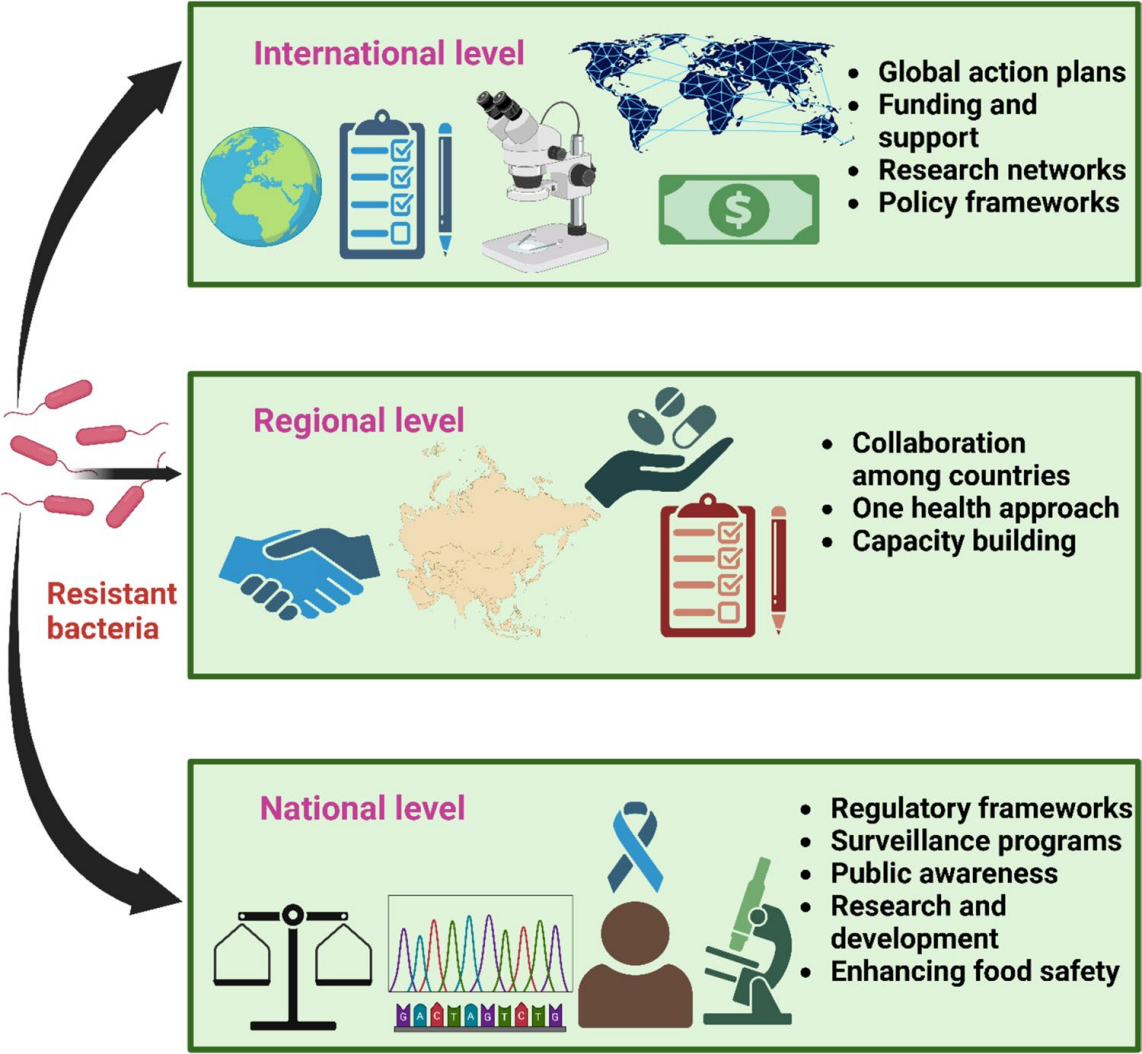
Combating antibiotic resistance requires a multifaceted approach at international, regional and national levels. Global action plans, funding to low and middle income countries, research collaborations, and policy frameworks to support international collaboration and standardized approaches are the main goals of global efforts to prevent antibiotic resistance. Regional collaborations focus on data sharing including joint surveillance and coordinated responses to outbreaks, a one health approach, and capacity building (training healthcare professionals and improving laboratory capacities) to collectively combat antibiotic resistance. At national level, countries are enforcing stricter antibiotic regulations, enhancing surveillance, raising public awareness, investing in research for novel alternatives and improving food safety to combat antibiotic resistance. This figure was created with BioRender. https://www.biorender.com/

Among the techniques that humans have innovated is the usage of modified or transgenic crops in order to minimize the use of antibiotics and develop a more effective strategy to resist diseases in crops. Genetically modified crops are those that have had a gene of your choice introduced, eliminated, or suppressed in order to generate plants with characteristics that are desirable (Grifths et al., 2005). To safeguard plants against insects and the infections causing microorganisms they carry, genetically modified crops that are resistant to insects have also been designed. Even though this boosts productivity of crops and relieves the monetary burden on cultivators, it is currently being observed that the population of target microbes or insects is becoming increasingly resistant (Bawa and Anilakumar, 2013). Policies at the national, regional, and international levels have been enacted lately as mentioned in (Fig. 3). While strategies for action differ immensely between countries and areas, they all seek to battle antimicrobial resistance (AMR) through the use of antibiotics cautiously, raising the concept of biosecurity and hygiene practices to avoid infections, and coming up with innovative antibiotic substitutes like probiotics, vaccines, and feed additives (Fig. 3). It's still debatable if these regulations and laws are effective to fight against antimicrobial resistance, the successful implementation of multifaceted synchronized initiatives can be measured by a number of vital factors (Kasimanickam et al., 2021).

Responsible antibiotic usage practices in farming and fisheries are crucial to prevent the development and spread of antibiotic resistance. This can be achieved by implementing good aquaculture practices, which lower disease risks and prevent disease, reducing the need for antibiotics and antimicrobials. The prudent use of veterinary medicines is also indispensable, as the use of antibiotics in animal rearing is important for increasing population survival rates, reducing chronic problems from infections, and improving food conversion rates. However, antimicrobials should only be used after careful consideration of the options for disease prevention and control (FAO). Project FMM/RAS/298/MUL: Consolidating capabilities, policies, and national action plans on the rational and judicious use of antimicrobials in fisheries was carried out by the FAO in 2017. The project's goals were to help participating competent authorities (CAs) build and implement national action plans (NAPs) and policies regarding the responsible and cautious use of antibiotics, as well as to improve their knowledge, abilities, and capacity in the areas of fisheries and aquaculture (FAO, 2020).

The US Food and Drug Administration (FDA) has been trying to encourage the use of medically required antibiotics wisely in animals that are the source of meat and milk. In order to protect public health, the FDA issued two non-binding guidelines for industry (GFI #209 as well as GFI #213) in the years 2012 and 2013, respectively (Apley, 2015). These instructions planned out the use of medically significant antimicrobial substances in food animals for production-related reasons (to boost growth or feed efficacy) while implementing their therapeutic uses (to cure, control, or prevent specific ailments) under the watchful eye of certified veterinary professionals (Apley, 2015; Ekakoro et al., 2019; Redding et al., 2020).

## Development and maintenance of a comprehensive surveillance system for antibiotic resistance

The U.S. food and drug administration (FDA) assembled a team of experts in 1994, and its members put forth the setting up of a national surveillance system aimed at tracking resistance among particular animal enteric bacteria that can cause diseases in humans. The panel of experts agreed and endorsed the foundation of National Antimicrobial Resistance Monitoring System (NARMS) in 1996 (Administration, 1994a, 1994b, 2001). State and municipal health organizations from every state in the union, in conjunction with three the federal government agencies—the CDC, FDA, and the US department of agriculture (USDA), cooperated to create NARMS (Karp et al., 2017). In the United States, microbes that frequently propagate through food are tracked for antibiotic resistance by the National Antimicrobial Resistance Monitoring System (NARMS) (Karp et al., 2017). Details on resistant to drugs intestinal bacteria, the dissemination of bacterial communities among individuals, animals used for food, and other sources, the genetic processes causing resistance, and the underlying causes and implications of resistant illnesses are all supplied by NARMS (Karp et al., 2017). These data are critical in establishing and evaluating the effectiveness of interventions designed to avert the emergence of resistance, as well as for guaranteeing that antibiotics approved for use in animals that are the source of meat, milk etc. are used in a way that is appropriate for the well-being of humans (Karp et al., 2017). The main objectives of NAMRS are:

i. Tracking enteric bacteria's resistance profile in human beings, commercial meats, and animals.
ii. To foster efforts that diminish resistance among foodborne bacteria, properly guide authorities in the USA and elsewhere concerning the development of antimicrobial resistance in infectious and commensal organisms.
iii. Investigate the development, prevalence, and transmission of antibiotic resistance by means of research.
iv. Share information that assists the FDA determine whether or not to approve effective and secure animal antimicrobial agents (FDA, 2021–2025).

The two main zoonotic pathogenic bacteria that cause food-related illnesses in the US, non typhoidal *Campylobacter* and *Salmonella,* are the focus of NARMS monitoring. The gut is the house of two most common bacteria, which are *Enterococcus* and *E. coli*, and they can be valuable sources of antibiotic resistance genes. In the gut, *Enterococcus* (gram positive) and *E. coli* (gram negative) get acted on by selective pressures that facilitate the development and maintenance of antibiotic resistance in these different bacterial groups. Thus, Gram-positive and Gram-negative bacteria each adapt to unique environmental challenges and pressures that cause their resistance mechanisms to evolve.” They are also included in food animal and meat sold in stores surveillance (Organization, 2017). NARMS investigated patterns of resistance in the food chain and identified new resistance risks via a holistic surveillance strategy. For instance, NARMS reported that non typhoidal *Salmonella* from retail meats, humans and food animals at slaughter, were growing more tolerant to ceftriaxone, a cephalosporin used to treat encroaching salmonellosis. The resistance fluctuates from source and serotype (Administrtion, 2011, 2012, 2015; CDC, 2014; Medalla et al., 2013).

In 2022 global antimicrobial resistance and use surveillance system (GLASS) reported resistance among prevalent bacterial pathogens. A major cause of concern is the median reported rates of 42% for third-generation cephalosporin-resistant *E. coli* and 35% for methicillin-resistant *S. aureus* across 76 nations (WHO, 2023a). In 2020, 1 in 5 cases of *E. coli* urinary tract infections showed decreased susceptibility to common antibiotics such as fluoroquinolones, ampicillin and co-trimoxazole. Additionally, it supervises the state of both new and old national surveillance systems, with a particular emphasis on the representativeness and quality of data assembling. Surveillance networks that enable GLASS enrollment and offer technical assistance to countries have been formed in some WHO regions (WHO, 2023a).

An integrated, unifying strategy known as "One Health" aspires to provide the best possible health outcomes for people, animals, and ecosystems in an ecofriendly manner (WHO, 2023a). It acknowledges the interdependence and robust relationship between domestic and wild animals, human health, plants, and ecosystem. Programs, legislation, policies, and research are designed, implemented, and monitored in collaboration with partners from relevant sectors under the one health approach to combat AMR and achieve better economic and health results (WHO, 2023a).

## Rapid detection and tracking of resistant pathogens in food products and the environment

For a long time, pathogens in food items have been characterized using culture-dependent approaches. These approaches are often straightforward to execute and cost-effective, but depend upon the pathogen being targeted, it can take up to three days for detection and seven days for confirmation of the specific pathogen. Furthermore, the detection of some pathogens that are not produced in culture, the capability to discern between culturable cells that are viable (living cells) and viable but non-culturable (VBNC) cells, and the dearth of adequate data to distinguish across strains are all downsides to culture-based approaches. The food sector has witnessed an upheaval in pathogen detection with the advent of nucleic acid amplification (NAA)-based technologies (Panwar et al., 2023). Next-generation sequencing (NGS) is one example of an advanced research approach that integrates bioinformatics and food science in order to fulfil the market demand for quickly and effectively detecting food pathogens (Liu et al., 2021). A variety of intriguing characteristics are provided by CRISPR-based technologies, including price effectiveness, convenient use, reliability analogous to PCR, many innovative detection platforms, and visualization methods for the sensitive and directed identification of pathogens. The CRISPR-Cas system is a configurable, versatile genome editing technology that uses the CRISPR RNA (crRNA)-guided Cas protein to cleave invading DNA and RNA. The "collateral effect" of RNase activity in a CRISPR-Cas 13a system can be elicited following the crRNA-mediated identification of the target RNA (Zhou et al., 2020). The upcoming era of foodborne pathogen identification with greater efficacy and experiment throughput rate is greatly projected with the integration of omics and CRISPR technological advances (Nehra et al., 2022). For foodborne pathogen, the biosensor beaded detection approach is straightforward, cost-effective, quick, and very selective. Traditional procedures, on the flip side, are laborious, slow, less precise, selective, and have poorer detection. Compared to traditional techniques, it is the most appropriate method for detecting foodborne pathogens because conventional methods cannot identify antimicrobial resistant pathogens that can enter the VBNC (viable but non-culturable) state such as *Vibrio cholerae* and *E. coli* from food (Kabiraz et al., 2023).

## Enhancing hygiene practices and food safety measures

A scientific course on food safety accentuates combating and preventing food-borne illnesses in all phases of the food production process, such as storing, handling, transporting, and preparing food, as well as guaranteeing the safety and health of food for human consumption. Being equipped with knowledge and competencies in food safety is the main objective of a scientific course. It aims at safeguarding public health and preserving the credibility of the food sector from farm to fork (Hashempour-Baltork et al., 2019). Antimicrobial resistant bacteria and their transfer from field to plate is significantly reduced by biosecurity measures in agricultural products and food items (Hashempour-Baltork et al., 2019). In 2011, the WHO and FAO defined biosecurity steps to reduce or entirely eradicate the likelihood of new diseases arising and propagating throughout a region or a nation. The primary focus of the actions known as critical control points and hazard analysis, good agricultural and veterinary practices, and good hygiene practices, is to manage microbes, assess risks, and ensure health. These practices aim to identify and control potential hazards in food production to ensure food safety. Thus, biosecurity can be economically beneficial to public health approaches, particularly for the agricultural sector where low chemical usage, well-managed farming, and good practices result in healthier animals and a decline in the need for antibacterial interventions (Hashempour-Baltork et al., 2019). In the regulation for industry guide to minimize microbial food safety hazards for fresh fruits and vegetables, the Food and Drug Administration (FDA) described the core principles of good agricultural practices (GAPs) (Jaysankar De, 2019). These guidelines offer extensive suggestions for lowering the risk of microbial contamination of fresh products to the fresh fruit and vegetable business. The US department of agriculture (USDA) formally launched the good agricultural practices & good handling practices (GAPs and GHPs) audit verification program in response to this guidance (Jaysankar De, 2019). These guidelines were put together to help local and international growers, packers, and shippers of fresh vegetables and fruits that have been partially or completely processed (but are still raw). In accordance to the FDA, these recommendations were wide-ranging and optional (Jaysankar De, 2019). Any complete food safety program should take into account the following GAPs assertions and clarifying remarks. The guidelines for water,municipal manure, sanitary facilities, sanitation of fields, worker health and hygiene, packaging facilities and transportation are summarized below as provided by Food and drug administration (FDA) [(FDA), 1998].

FDA guidelines emphasize the significance of preventing contamination through careful management of water, municipal bio-solids, and manure in order to preserve the safety of fresh produce. In order to prevent the introduction of harmful bacteria, water quality must be closely monitored. Contamination prevention is emphasized over the use of antimicrobials post-spoilage [(FDA), 1998]. In addition to appropriate application and handling procedures for manure, treatments like composting and ageing (storing manure or organic materials for extended periods (e.g. 6–12 months) are crucial for lowering pathogen risks associated with municipal bio solids and manure. Well-maintained sanitary facilities and handwashing stations are essential, and an effective action plan for accidents and leaks [(FDA), 1998]. Field sanitation involves cleaning equipment and storage facilities and preventing contamination of fresh produce from fertilizers, harvesting equipment, labourers and water. Worker health and hygiene are vital, requiring policies to manage sickness and proper training on hygiene practices. High standards of cleanliness must be upheld by packaging facilities, and in order to prevent microbiological contamination during produce transportation, meticulous vehicle inspections and adherence to sanitation guidelines are necessary [(FDA), 1998].

## Regulatory measures and policies

A systematic approach is used in the currently in force frameworks of regulation to deal with the potential hazards stemming from antimicrobial resistance (AMR) in the food chain in order to combat antibiotic resistance in foodborne microorganisms. These frameworks place an enormous value on the need of control, preventive, and surveillance tactics in the battle against AMR bacterial spread.

## Current regulatory frameworks

The noticeable characteristics of current regulatory frameworks consist of:

*Surveillance and risk analysis*: Executing risk analysis frameworks into practice to evaluate the risks associated with antimicrobial resistance (AMR) in food-borne pathogens and carrying out surveillance to track load of AMR bacteria in the food chain (FAO;WHO, 2023).

*Prevention strategies*: Stressing the significance of limiting the use of antibiotics in crop and livestock throughout the food production process in order to reduce the risk of AMR in humans. This involves evaluating the risk of introducing and spreading antimicrobial resistance (AMR) through viable microorganisms used in the production of food and feed crops (FAO;WHO, 2023).

*Control measures*: Creating and putting into practice national or regional recommendations for treatment that especially tackle AMR food safety issues, encouraging antimicrobial guidelines for responsible use, fostering infection control and biosecurity programmes to reduce the spread of resistant microorganisms from animals to human beings (FAO;WHO, 2023).

*International collaborations*: Addressing the global threat of antibiotic resistance requires working together with various organizations, partner countries, and international bodies like food and drug organization of the United Nations (FAO), world health organization (WHO), and the world organization for animal health (OIE). This encompasses pledging support for worldwide action plans and convening high-level gatherings to address AMR globally. The national action plan for combating antibiotic resistant bacteria of U.S, lays forth strategic objectives that are strategically integrated to fast-track the government's response to antimicrobial resistance and enhance public health for all citizens. The plan has pushed for radical shifts that bolster and exacerbate the response to threats of resistance. Pursuant to the national action plan, the US will collaborate globally as well as locally to prevent, identify, and cope with disease and mortality caused by illnesses as a result of antibiotic resistance. Beyond that, the code of practice to minimize and control foodborne antibiotic resistance was created by the codex alimentarius commission, a joint venture between FAO and WHO. This document offers evidence-based and risk-based recommendations for controlling foodborne antibiotic resistance and surveillance (CDC, 2020–2025).

The WHO approved the formation of a "strategic and technical advisory group" on AMR. International organizations suggested the one health strategy, which involves WHO, FAO, and OIE teaming forces to jointly establish a "tripartite alliance" in order alleviate the hazards associated with AMR. WHO also released a global action plan on AMR and started working with tripartite partners to resolve this global problem. The global foodborne infections network of the WHO and the international molecular subtyping network both use the colombian integrated programme for antimicrobial resistance surveillance (COIPARS) as a standard for foodborne infection surveillance. COIPARS techniques were employed by the advisory group on integrated surveillance of antimicrobial resistance by WHO to regulate AMR and the employment of antibiotics in food animals (Samtiya et al., 2022). At the 2015 World Health Assembly, nations agreed to address AMR globally by implementing national action plans that utilize a one health approach. These nations also endorsed the global action plan (GAP) on AMR. The world organization for animal health (WOAH), the governing bodies of FAO and the united nations environment programme subsequently acknowledged the GAP (WHO, 2023a).

## Various approaches and technologies that can be used as alternatives to antibiotics

**Fig. 4:**
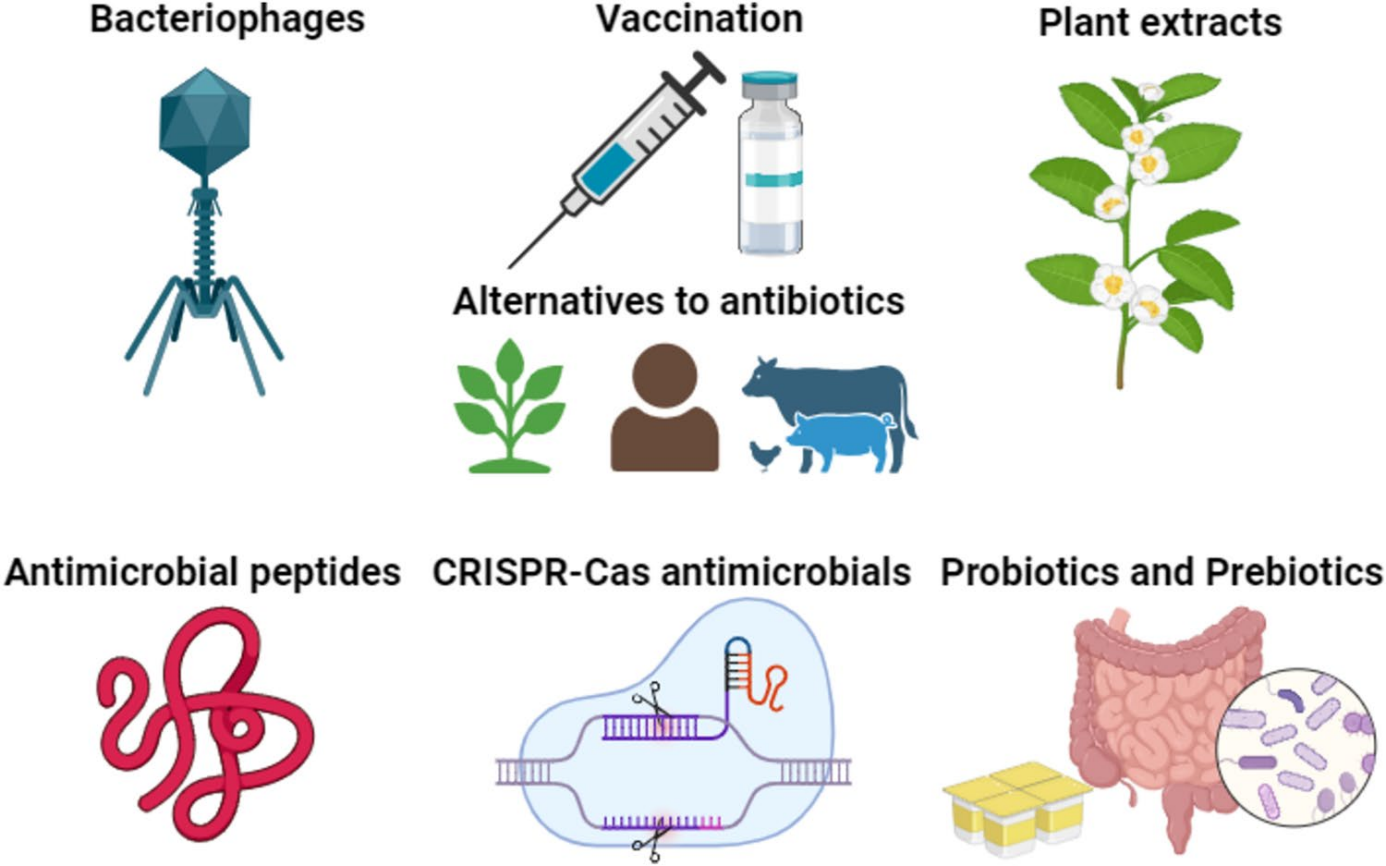
Alternatives to antibiotics. Phage therapy and bacteriophages, vaccination strategies, essential oils and plant extracts, antimicrobial peptides, probiotics and prebiotics, CRISPR-Cas antimicrobials are some of the alternatives that can be employed in agriculture and healthcare settings to combat antibiotic resistance. This figure was created using BioRender. https://www.biorender.com/

Given the ever-increasing resistance it is now crucial imperative to look for non-conventional anti-infective agents with novel mechanisms to kill microorganisms. Antimicrobial peptides (AMPs) have recently attracted extensive interest as potential therapeutic agents (Fig. 4) (Bin Hafeez et al., 2021; Ren et al., 2022). AMPs are distributed across all kingdoms of life and are an indispensable component of host defenses (Bin Hafeez et al., 2021). Most AMPs are oligopeptides of 5 to 100 amino acids with a positive net charge (typically + 2 to + 11) and a significant proportion (typically 50%) of hydrophobic residues (Bin Hafeez et al., 2021). In mammals, these proteins serve as the first line of shield against microbial invasion, while in prokaryotes, they are produced as a competitive strategy to impede the maturation of other microorganisms (Lei et al., 2019). In recognition of their antibacterial characteristics, AMPs are now widely employed in a variety of products as native alternatives to chemical additives for food safety and increasing shelf life (Jenssen et al., 2006; Lei et al., 2019). The reported mechanisms of action of AMPs are diverse and generally result in the direct killing of the pathogen, although several AMPs may also kill indirectly via modulating host immune responses (Bin Hafeez et al., 2021). An important feature that sets AMPs apart from conventional antibiotics is their attack on multiple low-affinity targets such as bacterial membranes, which is thought to mitigate the development of antimicrobial resistance (Bin Hafeez et al., 2021). A broad range of bacteria and archaea produce ribosomally synthesized peptide‒bacteriocins, with a length of 20–60 amino acids that are hydrophobic and cationic nature (Kumariya et al., 2019; Soltani et al., 2020). Bacteriocins have an appreciated impact on animal health and can be used to reduce or suppress infections, thus favoring livestock growth (Soltani et al., 2020). Pediocin PA-1 markedly accelerated the growth performance of broiler chickens infected with *Clostridium perfringens* (Soltani et al., 2020). The US Food and Drug Administration (USFDA) has approved the use of lactic acid, hydrogen peroxide, safe food preservation chemicals and certain peptides as biopreservatives (Soltani et al., 2020). Nisin is a most prominent bacteriocin produced by *Lactococcus lactis* that prevents the growth *of Clostridium* spp*.*, *S. aureus*, *L. monocytogenes,* and other bacteria and it is used for preservation of dairy items such as milk, canned vegetables, juice, alcoholic beverages, fish and meat (Soltani et al., 2020). Antibiotic resistance is a side effect associated with conventional antibiotic therapy that can be addressed with the use of nano-antibiotics. It has been demonstrated that silver nanoparticles (AgNPs) synthesized using *Caesalpinia sappan* extract are efficient antibacterial agents against methicillin-resistant *S. aureus* (MRSA) (Jun et al., 2015). One of the more promising uses of nanotechnology is nanoantibiotics, which uses the physicochemical conjugation of antibiotics with tiny particles or deliberately synthesized pure antibiotic molecules with a size range of at least 100 nm in one dimension (Mamun et al., 2021). Silver and ceramic nanoparticles, among other metal nanoparticles, are frequently used as nanoantibiotics, either alone or in conjunction with traditional antibiotics (Manjula and Chavadi, 2020).

Vaccination is an intriguing approach to avoid diseases and/or infections from occurring in people and animals (Fig. 4). It also reduces the need for antibiotics, which stops the growth and dissemination of bacteria that are resistant to treatment. The *H. influenza* type B vaccine and pneumococcal conjugate vaccines are two prominent examples of how successful vaccinations are at lowering AMR. Prior to the introduction of the polyvalent pneumococcal conjugate vaccination in the 1990s, there were over 63,000 cases of pneumococcal illness in the United States. As a result, *S. pneumoniae* developed a significant resistance to penicillin and other antibiotics. Following the vaccine's release, the disease's prevalence was drastically reduced and the pathogen's colonization was greatly reduced, both of which had a visible impact on resistant strains spread (Jansen and Anderson, 2018).

Antibiotic resistance is frequently linked to foodborne pathogens like *Salmonella* and *E. coli*, and these illnesses may originate due to the overuse of antibiotics, escalating the worldwide antibiotic resistance epidemic. Numerous vaccinations combating these infections are in their different developmental stages. For example, there are clinical trials underway for a 12-valent vaccine against extra-intestinal pathogenic *E. coli* (ExPEC). Vaccines against *N*. *gonorrhoeae* and *Salmonella enterica* ParaTyphi A are also in phase III of clinical trials (Mullins et al., 2023).

For numerous reasons, the development of vaccinations against prevalent foodborne pathogens, like enteric bacterial infections, continues to be extremely difficult. Research indicates that issues with security, efficacy, and long-term protective immunity have been encountered in the development of vaccinations against the main gut bacteria that cause enteric infections (Das et al., 2018). Additionally, the intricate nature of immune evasion mechanisms and microbial diversity presents difficulties in developing efficient vaccinations against infectious diseases caused by *Neisseria gonorrhoeae*, *Pseudomonas aeruginosa*, *and Staphylococcus aureus* (Microbiology, 2022). Scholars highlight the significance of emphasizing on various immunological mechanisms, such as T cell and antibody immunity, in order to boost the effectiveness of vaccines against these diseases. Targeting common foodborne infections with vaccines requires managing microbial diversity, complex immune responses, and the requirement for long-term protection. Sufficient research and developments in vaccination technologies are important to overcome these obstacles and improve food safety protocols. Any new vaccine takes a lengthened period of time to develop, typically a decade or two. Pharmaceutical companies should revamp the workflow to develop vaccines as a counteraction to the resistance issue. This may incorporate establishing new vaccination platforms and innovative technologies. It would also be essential to hasten clinical trials and alter how they communicate with regulatory bodies (Micoli et al., 2021).

The food and agriculture organization (FAO) of the UN has provided instructions on the principles and utilization of vaccines against diseases that affect cattle and poultry. The document emphasizes the benefits of livestock vaccination for rural livelihoods and the role it plays in preventing and controlling animal diseases (Metwally, 2022). An integrated federal-state-industry tool for managing certain poultry diseases is the national poultry improvement plan (NPIP), which offers immunization regimens tailored to the requirements of specific farms and regions (USDA, 2019). Multiple studies have shown that vaccinations against different bacteria and viruses may considerably decrease the amount of antibiotics used by animal populations. For instance, the use of antibiotics in the farmed salmon sector drastically dwindled after the introduction and regular use of a vaccine against *Aeromonas salmonicida* (Hoelzer et al., 2018).

Probiotics are live microorganisms that, when consumed in appropriate amounts, provide health benefits to the host (Bin Hafeez et al., 2024). Probiotics can be either allochthonous—which are typically not found in the flora of animal intestines—or autochthonous—that is, they can be either bacterial e.g. *Bacillus*, *Lactobacillus*, *Enterococcus*, and *Bifidobacterium* or non-bacterial (and fungal and yeast) (Afzal et al., 2020; Khan et al., 2021). Animals undergoing therapeutic treatment with antibiotics or other drugs may occasionally be given probiotics in order to allow for the repopulation or augmentation of gut flora that may have been compromised during the treatment (Fig. 4) (Rahman et al., 2022). The majority of research has shown that feeding probiotic strains to pigs and poultry, either in conjunction or alone substantially nurture feed conversion ratio (FCR), average daily feed intake (ADFI), and average daily gain (ADG) (Angelin and Kavitha, 2020; Rahman et al., 2022).

Prebiotics are substances that, by encouraging the growth of good bacteria in the gut, function as either food or fertilizer for these microorganisms. Prebiotics are a broad class of non-starch oligosaccharides or polysaccharides, notably lactulose, lactitol, fructans (inulin and fructo-oligosaccharides), mannan-oligosaccharide, galactans (galacto-oligosaccharide), gluco-oligosaccharides malto-oligosaccharide (Bin Hafeez et al., 2024). Beneficial bacteria in the large intestine ferment these nondigestible oligosaccharides, supplying the fuel to the microbiota (Rahman et al., 2022). Adhikari et al.2017 thoroughly studied and analyzed the function of prebiotic additives in enhancing growth, regulation of immune system, and pathogen cutback (Adhikari and Kim, 2017; Scott et al., 2020).

Moreover, studies revealed that predatory bacteria‒*Micavibrio aeruginosavorus*, *Bdellovibrio bacteriovorus*, associated organisms and two subgroups of proteobacteria can function as probiotics and antibiotics and can effectively treat ocular diseases in rabbits and cows caused by *Moraxella bovis* and *Shigella flexneri*, respectively (Hashempour-Baltork et al., 2019). Predatory bacteria like *Bdellovibrio bacteriovorus* target specific foodborne pathogens through their predatory nature, which allows them to effectively prey on a wide range of Gram-negative bacteria, including foodborne pathogens, without being affected by antibiotic resistance (Economou and Gousia, 2015).

Bacteriophages (phages) are viruses that infect bacteria. Their exceptional selectivity, lack of toxicity, and inherent abundance have made them quite popular in the last few years (Ranveer et al., 2024). Bacteriophages are distinctive in several ways. They do not leave residues in the ecosystem like chemicals or antibiotics do, they don't directly impact the microbiota like probiotics do, and they do not prioritize the immune response as vaccines do (Rahman et al., 2022). Bacteriophages primarily function as growth promoters through their antimicrobial activity since they have a very circumscribed range or spectrum of activity and only prey on particular troublesome strains without changing the microflora. Promising findings have been obtained from multiple researches on the application of bacteriophages to ward off pathogen infections in humans and animals (Fig. 4). For instance, a cocktail of four bacteriophages was introduced for use in on-farm therapy after it shown efficacy against isolates of human and bovine *E. coli* O157:H7. Furthermore, it was observed that bacteriophage biocontrol can lower the concentrations of *Campylobacter jejuni* in chickens without having an adverse effect on the gut microbiome. This can help avoid human exposure to infected poultry products and food-borne illnesses (Rahman et al., 2022).

Because of their antibacterial qualities, plant extracts and essential oils such as rosemary oil, cinnamon bark oil, tea tree oil, oregano oil, thyme oil and many others are being investigated more and more as potential antibiotic substitutes in agriculture (Fig. 4) (Bilal et al., 2020b; Nagore et al., 2021; Saeed et al., 2021; Shafiq et al., 2021; Shah et al., 2022). These organic substances have proven to be effective insecticides and crop protectors. Essential oils, comprise complex mixture of substances such as terpenes, alcohols, ethers, aldehydes, ketones, esters, amines, amides, phenols etc. that possess antimicrobial and antifungal potential (Abers et al., 2021; Alonso-Gato et al., 2021; Bashir et al., 2022; Lupia et al., 2024; Riaz et al., 2023). Cinnamon bark oil demonstrated antibacterial properties against *S. aureus* and *E. coli.* Oils like thyme, peppermint, cinnamon, oregano, and lemongrass are effective against *S. aureus* (Abers et al., 2021; Inouye et al., 2001; Abers et al., 2021 #350). Moreover, it has been suggested that plant secondary metabolites, such as essential oils, are safe and natural ways to treat bacterial infections in animals, which minimizes the need for antibiotics in animal husbandry. Essential oils' antibacterial activity is largely dependent on their chemical composition with key components like monoterpene hydrocarbons playing a significant role (Tanhaeian et al., 2020). Recent studies revealed that essential oils can be deployed as food preservatives and may even be able to halt the growth of bacteria that are resistant to multiple drugs (Hashempour-Baltork et al., 2019). Moreover, when used in conjunction with conventional antibiotics, certain essential oils can have a synergistic inhibitory effect that lowers the effective dose of the medications and reduces their side effects (Duarte et al., 2012). Fadli et al (2012) demonstrated that combining a traditional antibiotic with the essential oil of the endemic Moroccan thyme could have a synergistic effect in antimicrobial activity, leading to a decrease in the toxic side effects, necessary effective dose, and overall cost when it comes to drug-resistant bacteria (Fadli et al., 2012).

The use of CRISPR-Cas (an RNA-guided CRISPR system that targets DNA is called CRISPR/Cas12) technology to address antibiotic resistance in foodborne bacteria has demonstrated enormous potential (Fig. 4) (Qian et al., 2023). Certain diseases can be detected and eradicated by using the CRISPR-Cas system, a gene-editing instrument that targets the pathogen's DNA (Javed et al., 2023; Qian et al., 2023). It is theoretically possible to have an antibacterial effect in vivo by targeting and cleaving drug-resistant genes in drug-resistant bacteria, which would reinstate their sensitivity to antibiotics. This might be achieved by coupling CRISPR-Cas with phage delivery (Qian et al., 2023). CRISPR-Cas systems could be developed as "smart" antimicrobials to regulate the composition of gut microbiomes by eliminating antibiotic-resistant pathogens and halting spread of antibiotic resistance genes in medical applications, provided that the challenges with delivery and targeting efficiency are resolved (Duan et al., 2021). Promising techniques for identifying bacteria and their antibiotic resistance are engineered CRISPR-Cas systems, which have been shown to efficiently finish off bacteria or even reverse bacterial resistance to antibiotics. The dissemination of drug resistance genes, plasmid-mediated transformation and conjugation of antibiotic-sensitive organisms, and the transformation of plasmids carrying AMR genes can all be stopped by the CRISPR-Cas system. Multiplexed detection of AMR sequences, which is applied to assess the resistance of S. aureus strains and is crucial in the identification of MRSA infection vancomycin-resistant *E. faecalis*, is executed using a technology called FLASH (finding low abundance sequences by hybridization) that incorporates CRISPR-Cas (Wu et al., 2021). Some of the alternatives that can be used instead of antibiotics are represented in Fig. 4.

Nano pore sequencing (e.g., MinION), long-read sequencing (e.g., single-molecule real-time sequencing), along with short-read sequencing (e.g., Illumina MiSeq) are some of the methods offered by NGS, which is a versatile technology. The best approach will rely on the sequencing objectives because these techniques have variations in terms of accuracy, efficiency, and cost. With NGS, shorter fragments are integrated into a whole sequence, the completed genome is compared to reference strains, and bioinformatics techniques are applied to derive various conclusions, encompassing pathogen identification, high-resolution strain typing, and the forecast of significant phenotypic traits (Gwinn et al., 2019). When it pertains to public health, NGS provides major advantages for surveillance and epidemic investigation. These benefits include the ability to pinpoint clusters of connected cases more precisely, identify outbreaks earlier, and more promptly link illnesses to possible contaminated food sources. Compared to conventional techniques, NGS can recognize pathogens more quickly and precisely, offering new insights on the spread of disease, pathogenicity, and antibiotic resistance. Pathogen genome sequencing is being included into infectious disease surveillance by the US public health system of foodborne illnesses such as tuberculosis, hepatitis C, *Legionella*, and other pathogen which helps to accelerate the formation of more accurate, systematic and efficacious clinical laboratories (Gwinn et al., 2019). The next revolution in food safety diagnostics is the use of metagenomics-based techniques like shotgun and long-read metagenomics, which have the potential to directly identify entire microbial communities in a single food, ingredient, or environmental sample (Billington et al., 2022). Monitoring entire microbial communities as opposed to single pathogens offers a unique chance to increase our knowledge and capacity to manage microbial hazards, lower the prevalence of foodborne illness, and boost the economic sustainability and profitability of the food industry (Billington et al., 2022). Many bacteria might not be culturable or could be viable but not culturable; hence, a distinct selection and purification procedure is needed for each suspected species. By immediately detecting and characterizing complete microbial populations in a single food, component, or environmental sample in a single assay without the requirement for culturing, metagenomics techniques can overcome such limitations (Billington et al., 2022).

The food sector is impacted by nanotechnology, a fast emerging science, in a number of ways, including food processing, safety, and packaging (Bashir et al., 2022). Food quality can be enhanced, product shelf life can be extended, and food products can be tested for infections and poisons using nanotechnology. Additionally, it serves to create intelligent packaging and as an antibacterial in food packaging. The focus of nanotechnology techniques in food safety is on the antimicrobial characteristics of nanoparticles and nanosensors for the detection contaminants and foodborne pathogens (Mohammad et al., 2022). Compared to traditional approaches, nanotechnology offers the faster, more accurate, and more economical detection of pesticides, toxins, and other contaminants, as well as foodborne pathogens. These can be detected up by nanosensors at various phases of food production (Grumezescu and Holban, 2018). For instance, a study discovered revealed that 88% of *E. coli* could be isolated from a sample in 45 min using nanosized magnetic iron oxide particles combined with sugar molecules (Duncan, 2011). It was discovered that combining two or more nanoparticle materials produced a synergistic effect that led to a more effective antimicrobial than a single nanoparticle when it came to the antimicrobial properties of nanoparticles (Nile et al., 2020) For example, it was found that the combination of sliver nanoparticles with carbon nanotubes and titanium dioxide was twice as efficient against *Bacillus cereus* spores and *E. coli* (Mohammad et al., 2022). Food packaging use nanosensors to track temperature, time, and oxygen levels as well as identify contaminants, off-flavors, toxins, and spoilage (Mohammad et al., 2022) In addition to nanoparticles, there are many other uses for nanocomposites and nanolaminates in food packaging. They have been widely employed in the antimicrobial packaging of food products in recent years. Foodstuffs are primarily protected against mechanical and high temperature stunning by nano-composites, which also extends their shelf life (Honarvar et al., 2016). For instance, the food sector has paid a lot of attention to zinc oxide nanocomposite because of its antioxidant properties, which make it a popular choice for active food packaging (Kim et al., 2022). But there's a chance that nanoparticles could be hazardous to human health, so it's important to set up a suitable regulatory framework to control these risks (Mohammad et al., 2022).

## Public awareness and education campaigns about antibiotics and antibiotic resistance

In order to encourage responsible behaviour and reduce the growing threat of resistant infections, it is imperative that the public be made aware of the use of antibiotics and antibiotic resistance (AMR). Although most people have heard of antibiotics, not everyone is familiar with how they should be used. For instance, in a Singaporean survey, the majority of participants recognized the term ‘antibiotics,’ but only approximately one-third of them correctly identified that antibiotics work against bacterial diseases rather than viral ones, such as the flu or the common cold. In this survey, many respondents were aware of the term ‘antibiotic resistance’, though few knew the abbreviation ‘AMR’. While some understood that resistance involves bacteria becoming immune to antibiotics, a considerable number incorrectly believed it was due to the human body becoming resistant or antibiotics losing their power and effectiveness (Lim et al., 2021).

The World Health Organization (WHO) established "World Antibiotic Awareness Week" celebrated from 18 to 24 November every year, as part of its global action plan. In addition, the WHO published guidelines for a competent programme for health workers' training and education, as well as identifying gaps in the standard and analysis of initiatives to improve healthcare workers' AMR education and training (Samtiya et al., 2022). Mass media and social media frequently share messages on AMR-related issues that may reduce the incidence of AMR and antibiotic use, supporting awareness campaigns (Harbarth et al., 2015). Launched in 2018, the “superheroes against superbugs" (SaS) initiative aims to promote awareness, public and policy action on the issue of antimicrobial resistance (AMR) in order to address the growing threat of antibiotic-resistant infections in India (Superheroes Against Superbugs (SaS), 2020). Its primary goal was to collaborate with school children to creatively engage the public in conversations regarding AMR. The goal of these initiatives was to alter social norms, human behaviour, and the lack of knowledge about antibiotic usage and antimicrobial resistance (AMR) (Samtiya et al., 2022). In order to combat antibiotic resistance, the Health Education England Department, National Health Service of UK (HEE-NHS) has acknowledged the value of education and awareness and is promoting it at two levels: healthcare students’ education and community education, which includes school children, general public, and students pursuing careers in non-healthcare disciplines (President, 2014).

## Case studies

Netherlands and Denmark provide evidence of how laws and government involvement can increase animal productivity while reducing the use of antibiotics. In 1995 Denmark started to limit antibiotic growth promotion, and in 2000, the practice was outlawed (Parsonage et al., 2017). It wasn't until 2006 that the EU-wide prohibition was put into effect in the Netherlands. (Parsonage et al., 2017). Antimicrobial use in poultry decreased by 90% between 1995 and 2008 without evident loss in productivity (Parsonage et al., 2017). Danish pig production soared by 47% between 1992 and 2008 despite antimicrobial use declining by 51%. Between 2007 and 2012, the Netherlands had a 56 percent reduction in the use of antibiotics without any productivity losses (Parsonage et al., 2017). The Dutch experience implies that farmers changed their emphasis from using antibiotics to better management practices, avoiding ethical debates surrounding the use of antibiotics for accelerating growth. In fact, fewer Danish farmers are now operating, indicating that since the ban, only farms using ethical farming methods have been able to stay profitable (Parsonage et al., 2017). It is unquestionable that nations with adequate resources have managed to decrease the overall usage of antibiotics without compromising animal husbandry. On the flip side, analogous regulations in low-income nations can jeopardize less-than-ideal farming practices. There are arguments for the corresponding need to safeguard food production in developing nations, even in the face of opposition to a worldwide ban on growth promotion and prophylaxis (Parsonage et al., 2017).

## Challenges and limitations in addressing antibiotic resistance

**Fig. 5:**
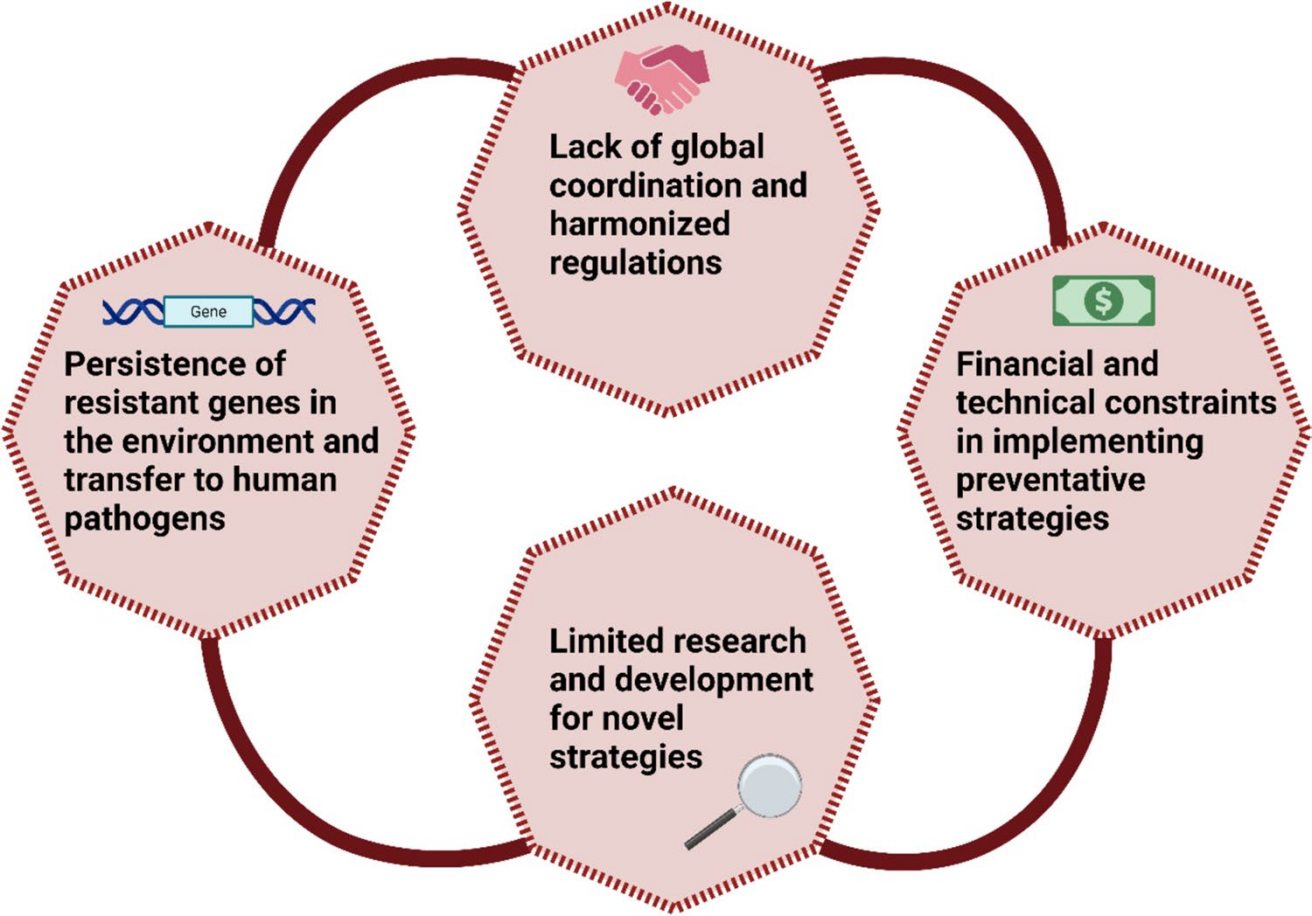
The challenges we are facing in combating antibiotic resistance. Lack of global and international regulations/policies or the failure to implement them properly, financial and technical obstructions in implementing preventative measures, limited or slender research and development for novel alternatives to antibiotics and the persistence of resistant genes in the environment through various evolutionary and ecological mechanisms are the major challenges in the battle against antibacterial resistance. This figure was created using BioRender. https://www.biorender.com/

There are several hurdles to overcome in combating and addressing antibiotic resistance as presented in (Fig. 5). The intricate framework of the food chain, which allows antibiotic-resistant pathogens to pass through the farm to store and harm both humans and animals, is a key contributor to this problem (Samtiya et al., 2022). One of the ruling impediment to tackling antibiotic resistance, according to the World Health Organization (WHO), is the lack of creativity in fostering the development of novel antibacterial medicines. According to the WHO's yearly pipeline report, the development of novel antibiotics to satisfy global demand is scarce, and the antibacterial clinical and preclinical pipeline is stagnant (Miethke et al., 2021; WHO, 2022). The burden of antibiotic resistance on morbidity and death rates has gone up due to the constrained availability of effective medicines prompted by inadequate progress in antibiotic research and development (D'Andrea et al., 2019). There is no one answer that can be found that does not lead to greater ethical concerns when it comes to the currently underway problem of controlling antimicrobial resistance. Every degree of ethical interpretation can be applied to the different aspects of medical practice and autonomy, prescribing, drug access, and economic position vs growing resistance. There needs to be more discussion about how to adopt global initiatives in order to continue the fight against resistance. Limiting antibiotic use to appease one stakeholder will almost likely have a later impact on the same stakeholder or a different one. Major challenges and limitations in the fight against antibiotic resistance are presented in **(**Fig. 5**)**. There is not a single approach that works for every AMR control, but there are some approaches that work straight away such as: infection control and prevention, greater incentives and investments for companies and academic institutions to mine new drugs and vaccines, including innovative methods to manage infections, enhanced global cooperation and accountability sharing, as well as broadened participation from nations and UN organizations to spur global intersectoral action on AMR, comprehensive public awareness campaigns, education and trainings to inform everyone about the possible depletion of effective antibiotics (Parsonage et al., 2017).

This article addresses the growing global issue of antibiotic resistance in foodborne pathogenic bacteria, examining trends in resistance, contributing factors, underlying mechanisms, and the consequences of this resistance. It also offers a comprehensive discussion of preventative strategies, emphasizing the importance of implementing measures such as improved food safety and farming practices, prudent use of antibiotics in veterinary medicine, alternatives to antibiotics, and enhanced surveillance to control the spread of antibiotic-resistant pathogens in the food chain. While significant progress has been made in understanding antimicrobial resistance (AMR) in foodborne pathogens, there remain several areas that require further exploration to effectively combat this global challenge. The application of advanced genomic and metagenomics techniques, such as whole genome sequencing, can provide deeper insights into resistance mechanisms, aid in surveillance, and help identify novel therapeutic targets. CRISPR-Cas9 gene editing, for example, can modify bacterial genomes to study the function of resistance genes and potentially disrupt those genes to reduce resistance. Additionally, advancements in microanalysis for resistance profiling, the development of DNA-based biosensors, and the use of machine learning and deep learning algorithms to detect DNA sequences associated with resistance genes are essential. The research and development of novel approaches, such as bacteriophages, synthetic biology, bacterial vaccines, natural antibiotics derived from plants and other organisms, and nanotechnology, should be prioritized and made more cost-effective and accessible. It is also a pressing priority to develop and modify regulatory frameworks to address the evolving threat of antibiotic resistance. Ensuring the strict implementation of these laws and regulations is crucial. Furthermore, we must escalate research and collaboration across various sectors and at the international level to develop innovative solutions to mitigate the threats associated with antibiotic resistance in foodborne pathogens and safeguard public health.
